# Gesture Decoding Using ECoG Signals from Human Sensorimotor Cortex: A Pilot Study

**DOI:** 10.1155/2017/3435686

**Published:** 2017-09-05

**Authors:** Yue Li, Shaomin Zhang, Yile Jin, Bangyu Cai, Marco Controzzi, Junming Zhu, Jianmin Zhang, Xiaoxiang Zheng

**Affiliations:** ^1^Qiushi Academy for Advanced Studies, Zhejiang University, Hangzhou, China; ^2^Department of Biomedical Engineering, Zhejiang University, Hangzhou, China; ^3^Key Laboratory of Biomedical Engineering of Ministry of Education, Zhejiang Provincial Key Laboratory of Cardio-Cerebral Vascular Detection Technology and Medicinal Effectiveness Appraisal, Hangzhou, China; ^4^The Biorobotics Institute, Scuola Superiore Sant'Anna, Pisa, Italy; ^5^Department of Neurosurgery, The Second Affiliated Hospital of Zhejiang University, Hangzhou, China

## Abstract

Electrocorticography (ECoG) has been demonstrated as a promising neural signal source for developing brain-machine interfaces (BMIs). However, many concerns about the disadvantages brought by large craniotomy for implanting the ECoG grid limit the clinical translation of ECoG-based BMIs. In this study, we collected clinical ECoG signals from the sensorimotor cortex of three epileptic participants when they performed hand gestures. The ECoG power spectrum in hybrid frequency bands was extracted to build a synchronous real-time BMI system. High decoding accuracy of the three gestures was achieved in both offline analysis (85.7%, 84.5%, and 69.7%) and online tests (80% and 82%, tested on two participants only). We found that the decoding performance was maintained even with a subset of channels selected by a greedy algorithm. More importantly, these selected channels were mostly distributed along the central sulcus and clustered in the area of 3 interelectrode squares. Our findings of the reduced and clustered distribution of ECoG channels further supported the feasibility of clinically implementing the ECoG-based BMI system for the control of hand gestures.

## 1. Introduction

Brain-machine interfaces (BMIs) have potential capabilities to bypass the interrupted motor pathways caused by neurological disorders or amputation and to build a direct communication between the brain and external devices by interpreting brain acitivities [1]. With this emerging technology, the paralyzed and amputated are able to perform simple actions through the external prosthesis and improve their ability of daily life [2].

Electrocorticography (ECoG) has been widely exploited to localize the seizure foci of the patients with intractable epilepsy for decades. Since ECoG signal could be collected through epidural or subdural electrodes placed on the surface of the cortex, it provides higher signal quality and spatial resolution than noninvasive neural signals, such as EEG signals [3]. Compared with neuronal ensemble recording, the implantation of ECoG recording is less invasive and reduces the clinical risks as well as ensures a long-term stability [4]. Therefore, ECoG has attracted considerable and extensive interest in BMI studies recently because of its good trade-off between performance and reduced invasiveness. ECoG has also been used to reconstruct high-dimensional arm movement [5, 6], predict movement directions [7] and single finger flexion [8–11], and classify hand gesture type [12, 13] as well as detect gross grasp movement [14, 15] with less training [16]. These studies demonstrate that the signal-to-noise ratio and the temporal-spatial resolution of the ECoG signals are sufficient to represent multiple hand gestures and provide commands to control a robotic hand in real time.

Although ECoG has been proven to be a good candidate of signal sources for BMI control, safety and reliability issues together in BMI field must be carefully considered before being applied into clinical practice. One of the main open challenges in this field is related to the minimization of the cortical area. Conventional intracranial ECoG electrodes are arranged in grids or strips covering a large area of the cortex surface aiming to simultaneously monitor brain regions and localize the seizure onset zone. Some studies pointed that the number and locations of electrodes which were chosen exclusively for clinical purposes did not exactly agree with the requirements in a BMI area. The large extent of the conventional implantation of ECoG grids exerts a great effect on the brain, which significantly increases the surgery and the postoperative recovery risks [17]. For these reasons, BMI users prefer a smaller but less invasive configuration of electrode grid without sacrificing decoding performance. By decreasing both the electrode diameter and the interelectrode distance, some studies customized micro-ECoG (4 mm center-to-center spacing) to realize a less invasive procedure and dectect movement intent with very local cortical activity. However, the optimal interelectrode distance is still uncertain and needs further study [18]. Beyond that, decreasing the number of channels on the premise of certain decoding accuracy could also reduce the area for grid covering. Different strategies have been proposed to optimize the number of ECoG electrode channels for the purpose of reducing the number of input features of the decoder and improve both decoding accuracy and computational speed. Among them, Milekovic et al. restricted electrode channels in a limited cortical region using neighboring channels to decode arm movement [19]. And Zhang et al. decoded visual stimuli using a single ECoG channel [20]. However, to our knowledge, few studies have investigated the optimal number of electrode channels in hand gesture discrimination and the corresponding anatomical distribution of these functional channels.

In this work, we aimed to analyze the ECoG representation in the sensorimotor cortex during the execution of hand gestures and reduce the area for electrode grid covering. The channels selected by single channel selection showed a cluster distribution during a hand gesture discrimination task and the greedy selection [21] was employed to further select the best electrode subset. The electrodes chosen by greedy selection were observed locating around the central sulcus and gathered in the area of three interelectrode squares. Notably, these selected electrodes were informative in distinguishing hand gesture types with high decoding performance in both offline and online real-time BMI system. Overall, these results contribute to our exploration of minimized invasiveness and help to further promote the clinical translation of BMIs into practice applications.

## 2. Materials and Methods

### 2.1. Participants and Implantation

All three participants in this study were suffering from intractable epilepsy and required surgical treatment for epileptic seizure control. The clinical subdural electrodes were surgically implanted in the sensorimotor cortex for clinical monitoring and localization of the seizure foci. The configuration and location of the electrodes, as well as the duration of the implantation, were determined by clinical requirements. The clinical electrodes were platinum electrodes with a diameter of 4 mm (2.3 mm exposed) spacing at 10 mm and generally implanted only for a period ranging from several days up to 2 weeks.The key information of the participants and their implantation sites are shown in Table 1.

All procedures were followed from the guide and approved by the Second Affiliated Hospital of Zhejiang University, China. Participants gave written informed consent after detailed explanation of the potential risks of the research experiment.

### 2.2. Cortical Mapping

Postoperative computed tomography (CT) scans were used to confirm the location of the electrodes. All three participants went through the clinical examination routine of the motor, sensory, language function, and so on through cortical stimulation mapping (CSM), which helped to further and functionally localize the electrodes. None of the hand motor areas of all the participants was seizure onset zone in our study.

### 2.3. Behavioral Tasks

Participants were instructed to perform one of three hand gestures (“scissors,” “rock,” and “paper”) or relax their hands in a rest position according to the cues presented on the screen in front of them.

**Figure 1:**
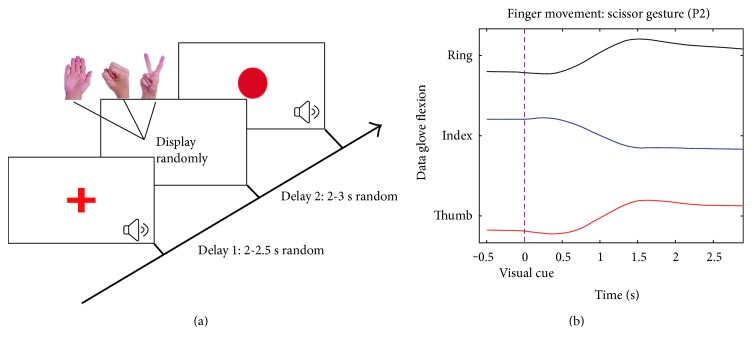
The behavioral task and recordings. (a) Hand grasp experimental paradigm. The trial was initiated by a red cross displayed on the center of the screen with a verbal cue “ready.” After a random delay ranging from 2 to 2.5 s, the red cross disappeared and the gesture cue appeared on the screen, indicating the participant to replicate the gesture shown and hold on it until the red dot came out 2-3 seconds later. The correction of the trial was fed back by another verbal cue at the end of the trial. Three types of the gestures were displayed randomly and equally. (b) The flexion of the data glove sensors on ring finger, index finger, and thumb. Purple dash line represented the timing when visual cue was displayed.

In the rest position, participants were asked to relax their task hands and flex the fingers slightly with palms facing up. A trial began with a verbal cue “ready” meanwhile a cross displayed on the center of the screen, indicating participants to keep task hands in the rest position and be prepared. This was the “baseline period” (2-2.5 s randomly). After the baseline period, the cross was replaced by a gesture picture (“go” cue (GC)), which randomly displayed one of the three gestures. Participants were informed to perform the gesture instantly and hold on it until a red circle (“stop” cue (SC)) appeared. The gesture displayed 2 to 3.5 s randomly. After SC, participants could release the gesture and return to the rest position. At the end of each trial, a verbal feedback, that is, “correct” or “wrong,” was given by the experimenter to inform the subjects whether it was a successful trial or not. The entire course of a task is illustrated in Figure 1(a). The trials were failed and excluded from the final dataset if participants were not able to hold on the gestures until SC appeared or forgot to release the gestures. Before the ECoG electrode implantation, participants were trained to acquaint themselves with the task until they fully understood the processes and requirements.

Each session was composed of 3 blocks, and each block was composed of 50 trials (5 sessions for P1, 5 sessions for P2, and 3 sessions for P3). Participants would have a short break between the blocks. In practice, the number of trials and the duration of each break depended on the medical condition and the willingness of the participants.

### 2.4. Neural Signals and Behavioral Data Recording

Clinical ECoG signals collected by subdural electrode grids were recorded by NeuroPort system (128 channels, Blackrock Microsystems, Salt Lake City, UT). The ECoG signals were firstly low filtered with a cutoff frequency of 500 Hz and stored continuously during the whole task at the sampling rate of 2 kHz. The channels which contained a high level of noise were excluded by visual inspection. The timestamps of external events, such as “go” cues and “stop” cues, were synchronized with recorded ECoG signals by acquiring timestamps from NeuroPort system. The behavioral data were collected by a 5DT data glove with 14 sensors (5DT Inc., USA). Each sensor simultaneously yielded flexion values for posture detection.  Figure 1(b) shows the flexion values of three sensors on ring, index, and thumb, respectively. The data were collected when P2 performed the scissor gesture, and the curves were smoothed by a Savitzky-Golay filter (3 orders, 101 points). We defined the occurrence of a movement onset when five first derivative of the flexion values consecutively exceeded a specific threshold.

### 2.5. Neural Signal Analysis

Offline data processing was performed on a MATLAB platform (Natick, MA). First, a spatial filter, that is, common average reference, was applied to all the remaining channels after visual inspection to remove common noise.

#### 2.5.1. Feature Extraction

ECoG feature was the power spectrum captured in different frequency ranges. By analyzing its dynamical spatiotemporal pattern, we could characterize the neural features associated with different movement status and hand gesture types. To obtain the time-resolved power spectrum of ECoG signals, the ECoG time series of an entire session were segmented into 300 ms width windows with an overlap of 200 ms. Then, a 3 order multitaper spectral estimation was employed to calculate the power spectrum $\hat{S}\left(f,T\right)\;$in each individual window of all the selected channels. Specifically, this approach applys a set of orthogonal tapers: *a*_*k*_(*t*), *k* = 1,…, *K* to time series and minimizes variance by averaging all the tapered and independent spectra:

(1) $$\begin{array}{c} {\hat{S}}_{k}\left(f,T\right)={\left|\overset{N-1}{\underset{t=0}{\sum }}x\left(t\right)a_{k}\left(t\right)e^{-2\pi jft}\right|}^{2}, \end{array}$$

where *N* is the length of *x*(*t*) and  *T* = *T*_1_,…, *T*_*m*_ denotes the time index corresponding to each 300 ms window. Then, the averaged spectrum is given:

(2) $$\begin{array}{c} \overset{-}{S}\left(f,T\right)=\frac{1}{K}\overset{K}{\underset{k=1}{\sum }}{\hat{S}}_{k}\left(f,T\right). \end{array}$$

Whereas due to the fact that the power spectrum of brain signals decreases with increasing frequency, known as a “power law,” the changes in low frequency will dominate over the whole frequency range. A normalization in each frequency bin of the spectrum is very necessary to eliminate this phenomenon for the observation of the power spectrum variation in high frequency. Therefore, baseline power spectrum *S*_baseline_(*f*) was calculated by averaging the power spectrum obtained during the baseline period before the visual cue across trials. Then, the frequency-resolved power spectrum *S*_norm_(*f*, *T*) during the period of task execution was normalized by dividing averaged baseline power spectrum:

(3) $$\begin{array}{c} S_{norm}\left(f,T\right)=\frac{\overset{-}{S}\left(f,T\right)}{S_{baseline}\left(f\right)}. \end{array}$$

#### 2.5.2. Decoding

**Figure 2:**
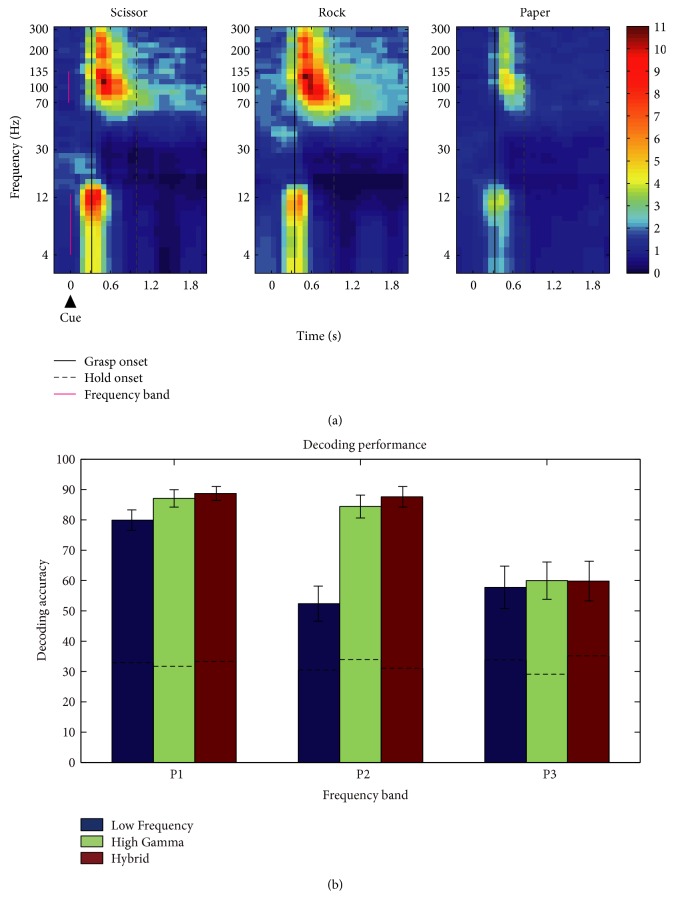
Time frequency plots of the ECoG signals and decoding performance obtained from all channels and selected channels. (a) Normalized power spectrum of ECoG signal from a representative channel 35 over sensorimotor cortex of P1 which was averaged and aligned with visual cue (time = 0). The frequency increases in log scale, and the color bar gives the scale of the spectrum amplitude values. The gestures from left to right are scissor, rock, and paper, respectively. Vertical magenta bars in the subplot indicate the frequency bands used in decoding (high gamma frequency band (70–135 Hz) and low-frequency band (4–12 Hz)). Black solid lines represent the averaged grasping onset across trials, and the grey dash lines represent the averaged end timing of grasping. (b) The decoding performance of different frequency bands (low-, high-, and hybrid frequency band, from left to right) using all channels (left column). The performances of hybrid frequency band are significantly higher than that of high frequency band in P1 and P2 (*p* value < 0.01), and both the hybrid and high frequency decoding results are higher than low frequency in all participants. The black dash line represent the chance level.

We used the Matlab Libsvm package to build a multiclass SVM classification model and realize gesture type classification [22]. The input features of this classifier were the frequency-resolved power spectrum across specific frequency bins, time bins, and channels. The corresponding target outputs were the labels of gestures (“scissors” = 1, “rock” = 2, and “paper” = 3). Among them, the frequency bins = frequency band width/frequency resolution. And time bins spanned from [*T*, *T* + Δ*T*,…, *T* + 9Δ*T*], in which *T* was the time of movement onset and Δ*T* was one time bin at the time step of 100 ms. We chose 10 time bins for the reason that both high gamma frequency band (70–135 Hz) and low-frequency band (4–12 Hz) were observed highly active during this period as shown in Figure 2(). Therefore, the features formed a 3-dimensional matrix, where channels, frequency bins, and time were the three dimensions. The final feature of each trial was later reshaped into a 1*∗n* (*n* = number of channels*∗*frequency bins*∗*10 time bins) vector.

In offline decoding, we pooled all the trials of each individual participant and applied a 3-fold cross validation to the data set. The decoding performance was the average of the percentage of correct predictions using testing data 50 times. Besides, the chance level was the result of the 95th percentile of the decoding accuracy distribution which contained 10,000 results generated from the testing data with randomly shuffled labels. We used a *t*-test as the significance test and calculated the *p* values.

#### 2.5.3. Channel Selection Strategies

We used the single channel selection and the greedy selection to progressively select the *n* best channels offline in our study.

In the single channel selection, we first calculated the decoding performance of each individual channel. The *n* best channels were the channels achieving the *n* highest decoding performances on single channel level, constituting the input vector of SVM for training and testing.

In the greedy selection, we first picked out the channel which yielded the best single channel performance from the total number of channels. Then, in the next round, the second best channel was selected which could improve the decoding performance most when paired with the first channel. The *n* best channels were selected successively by repeating the process and then constituted the input vector of SVM for training and prediction. The decoding performance saturated with *n* channels when there was no significant increase in decoding performance using *n* + 1 best channels.

In this study, we also compared the decoding performance using most neighboring 4 and 9 channels.

### 2.6. Real-Time Prosthetic Hand Control

The last sessions of P1 and P2 were used to evaluate the real-time performance of this ECoG-based prosthesis control system. The first two blocks were used to train the SVM decoder model, and the last block (50 trials) to evaluate the performance. This system managed to extract the neural signal data every 100 ms from the buffer of the neural signal processing and compute the features immediately at the time point receiving the visual cue. Codes were written in C language. The features were normalized by the baseline power in training data set then translated by SVM classifier into one of the three gesture types without cross-validation. Finally, gesture type was interpreted into control commands sent to an artificial hand through a serial port. The artificial hand stood by until the commands arrived and then executed the corresponding gesture in one second. This artificial hand was designed to duplicate the human hand movements with 6 DC which drive six degrees of freedom in the artificial hand (five fingers and the wrist).

## 3. Results

**Figure 3:**
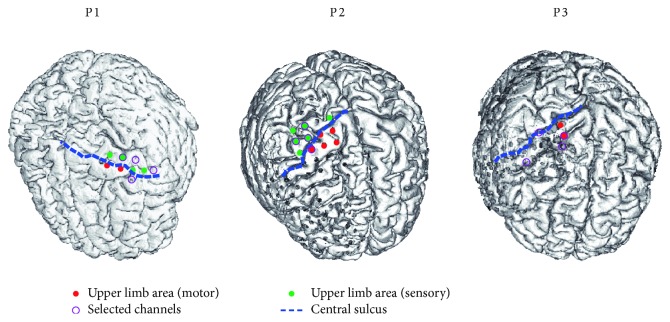
The locations of the subdural ECoG electrodes and their results of cortical electrical stimulation. Red dots indicate the locations of electrodes over primary motor cortex. Green dots indicate the locations of upper limb areas over primary sensory cortex. Purple circles represent the channels selected by greedy algorithm (see Channel Selections and Anatomical Patterns). Blue dash lines mark the central sulcus.

The data set analyzed in this study was collected from three participants (see Materials and Methods). The trials were selected only when they successfully met the task requirements stated in the Materials and Methods.  Figure 3() shows the locations of each electrode grid on the cortical surface which were confirmed by the postoperative CT scans.

### 3.1. Time-Frequency Analysis and Decoding Performance

The time-frequency plots illustrating the normalized and averaged power spectrum of three different gestures recorded by one representative channel of P1 are illustrated in Figure 2(a). The time-frequency plots were aligned with the “go” cue. The power spectrum of ECoG signals showed a movement-related modulation during hand movement. The power increased from the movement beginning to the end in both high gamma frequency band (>70–135 Hz) and low-frequency band including theta frequency band (4–8 Hz) and alpha frequency band (8–12 Hz). This task-related modulation existed in similar frequency bands (high gamma and low-frequency bands) across participants. However, the modulation patterns of the power spectrum in these frequency bands varied with different gesture types, indicating that the modulation patterns might contain exclusive information of each gesture type. Therefore, it was highly possible to distinguish the gesture types using power modulation patterns in specified high gamma and low-frequency bands.

The decoding performances of all three participants are shown in Figure 2(b). ECoG features were extracted from low-, high gamma, and hybrid frequency bands of all channels, respectively. The offline decoding performances of all participants in different frequency bands were significantly above the chance level, and the performance could reach to nearly 90% of both P1 and P2. The hybrid decoding results significantly outperformed the other two frequency bands, and high-frequency decoding results significantly outperformed the low-frequency performance in all the conditions except the performance of all channels in P3 (*t*-test, *p* < 0.01).

### 3.2. Channel Selections and Anatomical Patterns

**Figure 4:**
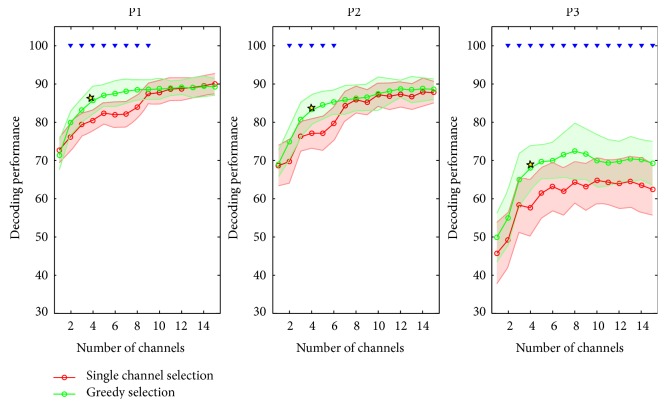
The decoding performance varies as a function of the number of channels. The green curves represent the decoding performance using the channels selected by greedy selection, and the red curves represent the result of the channels selected by single channel selection. The performance of greedy selection significantly yields higher performance than that of single channel selection when the same number of channels was used. The upside-down triangles indicate that the greedy selection performance is significantly higher than the single channel selection performance. The yellow stars indicate that the decoding performance obtained from the channel subset reaches the saturated point in the greedy selection. No significant improvement of decoding performance was found after adding more channels into the subset.

**Figure 5:**
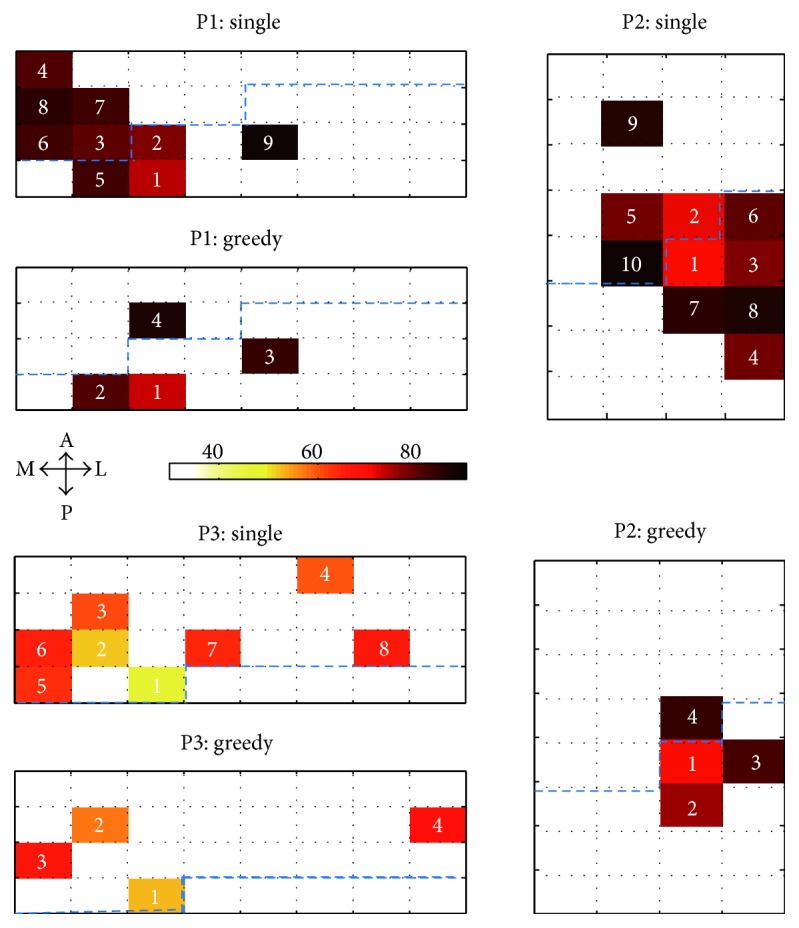
The spatial distributions of the selected channels using single channel selection and greedy selection of all three participants. The color bars give the scale of decoding performance. The numbers on the grid indicate the order of the selection. The performance yielded when the current channel was added into the selected decoding channel subset. All the ECoG grids are in 4*∗*8 configuration. Blue dash lines mark the central sulcus.

In this study, the single channel selection was first employed to intuitively select the channels with highest decoding accuracy (see Materials and Methods).  Figure 4 plots the decoding performances varied with channel numbers based on single channel selection, and they are saturated with 9, 10, and 8 channels in each respective participant. It could be found that using single channel selection, many plateau points occur before the decoding performance reached to a saturated point, indicating that there is some movement-related information redundancy among these selected channels.  Figure 5 maps these selected channels to the implanted electrode grids. Except for a few channels, the general distributions of these channels present a pattern which can be categorized as clustered.

To reduce the redundancy brought about by single channel selection and select the most contributive channels, we further tried the greedy selection. Decoding performance of all the participants displayed a trend of fast increase at the beginning and then saturated after a certain number of channels were added as shown in Figure 4 using greedy selection. Notably, the decoding performance using four best channels selected by the greedy algorithm reached to their saturated points in all three participants, which were 85.7%, 84.5%, and 69.7%, respectively. Although their performances were slightly lower than those obtained from all the channels, the decoding performances were no longer significantly improved when much more additional channels were added into the subsets. Furthermore, the greedy selection showed significantly higher and stable performance than the single channel selection algorithm did when the same number of channels was used for the neural decoding. It inferred that the greedy selection algorithm was much more appropriate and effective for selecting the minimal number of channels than the single channel selection. Furthermore, we found that most of these selected channels are clustered together and distributed along the central sulcus (shown in Figure 5). For P1 and P2, the selected channels situated mainly in the precentral gyrus except one in the postcentral gyrus. All of the channels were next or very close to the central sulcus at the maximal distance of two electrodes spacing. For P3, all selected channels were located at the site of precentral gyrus and group together except one channel away from the others.

### 3.3. Decoding Performance Using Nearest Neighboring Channels

**Figure 6:**
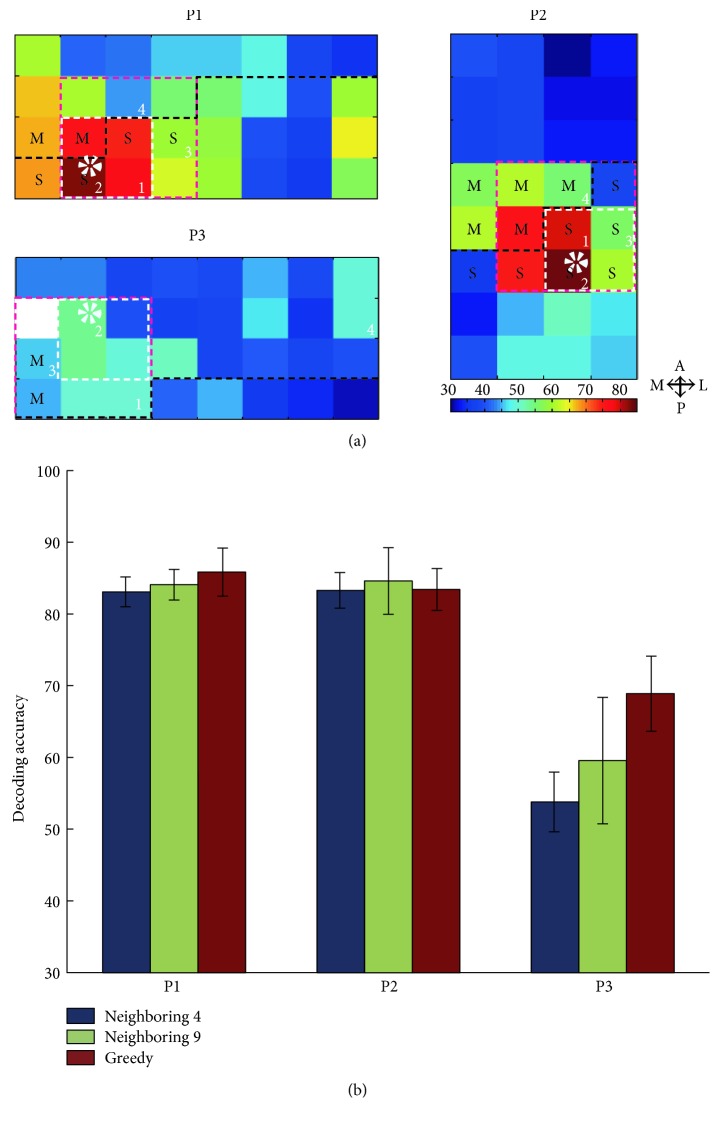
Topographies of decoding performance using neighboring 4 channels and the decoding performance comparison of different channel selection strategies (neighboring 4, neighboring 9, and greedy) of all participants. (a) The color of each cell indicates the decoding performance using the recordings from neighboring 4 channels. The color bars give the scale of decoding performance. The white dashed outline marks out the channels with the highest decoding performance (marked with a white dashed circle), and the red dashed outline marks out the neighboring 9 channels. Black dash line represents the central sulcus. (M = motor, S = sensory). (b) The decoding performance with optimal channel subsets using different channel selection strategies of all the three participants.

In the above results from all three participants, we found that the saturated decoding performance could be achieved by only a few number of the best channels selected by the greedy algorithm. This saturated performance is very close to that obtained with the neural signals from all channels. It is worth noting that the best three or four channels selected by the greedy algorithm are spatially close to each other and most of them were located near to the central sulcus. It suggested that the ECoG signals from these neighboring channels along the central sulcus might contain the most information about the difference between three types of gestures. Therefore, we tried to investigate whether such a subset of neighboring electrodes from a small subregion of ECoG grid could provide enough neural signals for distinguishing three gestures by evaluating the decoding performance of the nearest neighboring 4 and 9 channels.  Figure 6(a) illustrates the highest decoding performances with different channel selection strategies (neighboring 4, neighboring 9, and greedy algorithm). All three strategies for channel selecting achieved promising performance, which was significantly higher than the chance level. In P1 and P2, the decoding performances of three strategies were all above 82% and very close to each other. No significant difference was found among three strategies in P2. Although the decoding performance of the greedy algorithm is significantly higher than that of the neighboring 4 selection in P1, the difference between them was less than 4.0%. A general spatial pattern emerges, as Figure 6(b) shows that most of the channels selected by the greedy algorithm and by the neighboring 4 channels are also included in the square of optimal neighboring 9 channels and the channels are distributed along the central sulcus.

The results of both greedy and neighboring channel strategies consistently demonstrate here that the channels for hand gesture decoding could be restricted to the area of 3∗3 square (approximately 4 cm∗4 cm) when using clinical subdural electrodes with a high decoding accuracy.

### 3.4. Synchronous Real-Time Prosthetic Hand Control

**Figure 7:**
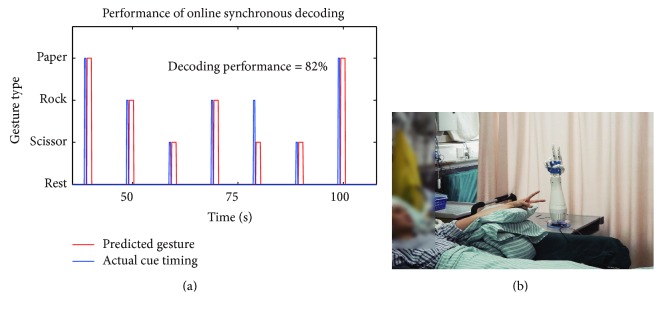
Real-time ECoG-based synchronous gesture types decoding performance. (a) An epoch of the real-time prosthetic hand control state of P1. Red line is the predicted gesture state, and the blue line is the actual visual cue timing. The real-time decoding performance of that session is 82%. (b) The photo taken at the experiment scene.

In order to further assess the feasibility of this online grasping BMI, we applied the real-time ECoG-based control system using an artificial hand to P1 and P2. The system collected the neural data every 100 ms from the Neuroport System buffer, extracted, and decoded features in real time on a personal computer (see Materials and Methods). A SVM classifier with the selected channel subset, hybrid frequency bands, and time lag learned from the offline analysis was trained using the first two blocks in that session and predicted gesture type in the last block. Classification results were interpreted into commands and sent to the artificial hand by a serial port communication protocol. A representative epoch of the prosthetic hand status of P1 containing 7 trials is depicted in Figure 7. The decoding accuracy in that entire block was 82% (41/50). And the decoding accuracy in P2 achieved 80% (40/50).

## 4. Discussion

Clinical ECoG signals from the sensorimotor cortex have a good temporal and spatial resolution to represent and discriminate hand gesture types. However, instead of long-term chronic implantation, most of the existing ECoG electrode grids for signal recording are customized mainly for epileptic focus localization and implanted temporarily. In the current study, satisfying decoding performance could be achieved by only a few and clustered channels from a small area rather than all channels on the grid in all three participants. And a real-time ECoG-based prosthesis control system was implemented with a small subset of channels.

Many previous studies have demonstrated that high gesture decoding performance could be achieved by using ECoG signals [12–14]; only a few of them considered the possibility of striking a balance between decoding performance and the number of channels when decoding hand gesture types.

In the 2D arm movement decoding, Milekovic et al. [19] tried the channel selection strategy of paired channels, neighboring three or four channels to search for the maximum decoding accuracy in a subregion of grids. They pointed out that it was sufficient to extract and decode the movement information with the recorded neural signals only from a rather small relevant cortical region.

In this study, we first examined the relationship between the decoding performance and the number of channels for decoding in two channel-selecting strategies. The decoding curves of the single channel selection and the greedy selection both show a rising beginning. Then, the decoding performances were saturated after a small subset of channels was progressively added. These trends are significantly obvious for the greedy algorithm in all three participants. Our results demonstrated that in the hand gesture decoding, a small subset of channels could also achieve high decoding performance close to that of all channels.

To further examine the spatial layout of the channels selected by two strategies, we mapped the channels to the electrode grids placed on cortical surface. A general spatial layout emerged that the selected channels clustered in a small group and distributed along the central sulcus. Our results are consistent with the findings of Chao et al. [4] who also found that the best decoding performance of muscle activity and that of the hand trajectory are efficiently generated by the electrodes close to the central sulcus. This result also supports the findings of Milekovic et al. and expands their application to the hand gesture decoding by using ECoG signals from a rather small relevant cortical region.

Furthermore, we found that most of the selected channels were distributed along the side of the postcentral gyrus in two participants. Pistohl et al., Chestek et al., and Wang et al. also showed that high classification of gesture types could be obtained from the channels on the postcentral gyrus [12, 15, 23]. It is worth noting that Wang et al. managed to decode 3D arm movement with the ECoG signals obtained from the postcentral gyrus of a paralyzed participant, who cannot move his limbs at all [24]. Therefore, it suggested that the activation of the postcentral gyrus played an influential role in hand movement. This phenomenon is probably due to the motor control copy or the force-related feedback. Moreover, we found that there were some adhesions between the dura and skull above the primary sensory cortex in P3, which brought difficulties in implanting the electrode grid. Therefore, no electrodes were located in the postcentral sulcus. It was also observed that the quality of the neural signals was much noisier than those of the other two. We suspected these above-mentioned facts led to inferior decoding performance of P3. Besides, previous researchers showed that the activated cortex of the sensory-motor area was clustered during the movement of different fingers and wrist. But this kind of grouping tend to be varied with the movement types. Our results showed that during the hand gesture movement, the selected channels were clustered along the central sulcus, especially in the postcentral area. But further investigation was required to understand whether it also applies to other complex movement tasks.

We also observed that the decoding results of the selected channels (P1: 85.7%, P2:84.5%, and P3:69.7%) were slightly lower than the results of using all the channels in P1 (88.7%) and P2 (87.6%) and even significantly higher in P3 (59.8%). The small channel subset had a good approximation of the performance obtained with all channels (shown in Figures 2(b) and 6(b)) using greedy selection. These differences might be related to the fact that instead of calculating all the possible combinations to search for a global optimum, the greedy algorithm yields local optimum at each stage. In this study, we used greedy selection and took the decoding performance of the SVM-decoder as the evaluation criteria to select clustered channels and minimize the electrode covering. In terms of greedy selection, this method works faster and implements easier than other optimization methods for no exhaustive operation on all data was needed. However, as far as multiclass SVM was concerned, more decoder models could be employed and investigated to reduce the dimensions of feature space for further optimizing the decoding performance, such as sparse SVM. Most importantly, the number of electrodes could be reduced from 32 to 4 and the region could be restricted into a much smaller area of 4 cm∗4 cm. This investigation could further benefit the development of wireless Bluetooth devices for neural signal transmission.

Features were extracted from hybrid frequency bands (4–8 Hz, 8–12 Hz, and 70–135 Hz) in this study. The hybrid frequency bands yielded higher decoding accuracies than either high- or low-frequency band in both P1 and P2, indicating that both low- and high-frequency bands contained different movement-related information. It was also found that the high-frequency band significantly outperformed low-frequency band in all participants, which suggested that high frequency played a much more important role in hand gesture classification. However, the decoding performance in high gamma band dropped dramatically in P3. This result might also be explained by the much noisier signals of P3 since the high-frequency signal was much more sensitive to the noise. As shown in Figure 2(a), the movement-related frequency range was not just limited to the selected frequency range. The frequency range could extend to 200 Hz in P2 and P3 and even up to 300 Hz in P1, consistent with the characteristic of board band in the previous research findings [25]. Although the ultrahigh frequency bands are not included in our decoding system, they might contain additional useful information and be useful to decode the finer hand movements. On the other hand, the beta frequency band ranging from 12 to 30 Hz shows a movement-related power suppression in our study. Although this band is less specific to hand gesture types as previous studies showed [13], its movement-related activation could be potentially employed in movement onset detection. The further and detailed analysis on the movement-related features would help to design a more advanced BMI. In addition to the features in frequency domain above, the features in the time domain, such as local motor potential [6] or simply averaged ECoG, have been widely supported by accumulated evidence that they might be promising neural features in BMI. It is worth for further examinations before they are applied to the ECoG-based prosthetic hand control.

Additionally, as displayed in Figure 3, the selected channels were all located inferior to the corresponding functional channels identified by cortical stimulations. One possible reason might be related to that the electrical stimulation which was conducted for sensorimotor localization was coarse during the examination. In addition, the channels for hand gesture decoding could be restricted to the area of 4 cm*∗*4 cm. And other documents showed that the edges of the hand-related area including palm and five fingers in the somatosensory cortex also spanned approximately 4 cm. However, more researches are needed to investigate whether the area of selected channels consistently corresponds to some functional regions across participants. Many functional neuroimaging techniques could be adopted, such as functional magnetic resonance imaging and magnetoencephalography, to precisely localize the placement of ECoG electrode grids and the projected fields of each functional electrodes.

Finally, we have shown that the synchronous hand gestures could be decoded using an ECoG-based real-time control. However, when applied it into daily life, it might be unnatural because most of the movements are controlled by voluntary modulations without a particular hint at a particular “go” cue [24]. Therefore, in the future, an asynchronously BMI which starts or releases at will might be more feasible and suitable to realize a natural and practical hand movement. Currently, some of the researchers found that the grasp intentions were represented by certain neural activity patterns. They also proved that this intention could be detected from the sensorimotor cortex [23]. These studies were only limited to few participants and it is unclear whether it is the same to multiple hand gestures classification. Furthermore, theoretically, the release of the gesture could also be detected for it is another kind of hand-shaping movements, but in practice, it needs more careful examinations for distinguishing it from the neural activity in a hand-shaping period.

## 5. Conclusion

In this study, we explored the feasibility of implementing an ECoG-based BMI which could decode real-time three hand gestures and control an artificial hand. Three participants with ECoG electrode grids placed over the sensorimotor cortex were involved. To achieve minimal craniotomy, several strategies were employed to select the subset of channels. A general spatial distribution of these selected channels was showed as clustered along the center sulcus. With the selected channels, high decoding performance was maintained. It is expected that this results would help to translate BMI application towards practical and clinical realization.

## Figures and Tables

**Table 1:** Participant overview and grid location.

Participant	Gender	Age	Handedness (task hand)	Implanted grids	Seizure onset zone
P1	Female	28	Right (right)	Left hemisphere: temporal, parietal, occipital lobe	Anterior temporal lobe
P2	Male	22	Right (left)	Right hemisphere: frontal medial, dorsal surface, parietal lobe	Anterior frontal lobe
P3	Male	22	Right (left)	Right hemisphere: temporal, occipital lobe	Right temporal lobe
